# A systematic review of the impact of postoperative aerobic exercise training in patients undergoing surgery for intra-abdominal cancers

**DOI:** 10.1007/s10151-023-02844-9

**Published:** 2023-08-07

**Authors:** M. Paul, T. F. Smart, B. Doleman, S. Toft, J. P. Williams, J. N. Lund, B. E. Phillips

**Affiliations:** 1 Centre of Metabolism, Ageing and Physiology (COMAP), School of Medicine, MRC-Versus Arthritis Centre for Musculoskeletal Ageing Research and National Institute of Health Research (NIHR) Nottingham Biomedical Research Centre (BRC), Academic Unit of Injury, Rehabilitation, and Inflammation Sciences, University of Nottingham, Royal Derby Hospital Centre, Derby, DE22 3DT UK; 2 Department of Surgery and Anaesthetics, Royal Derby Hospital, Derby, UK; 3 Library and Knowledge Service, University Hospitals of Derby & Burton NHS Foundation Trust, Derby, UK

**Keywords:** Exercise, Cancer, Surgery, Fitness, Rehabilitation

## Abstract

**Introduction:**

Enhanced recovery after surgery (ERAS) programmes which advocate early mobility after surgery have improved immediate clinical outcomes for patients undergoing abdominal cancer resections with curative intent. However, the impact of continued physical activity on patient-related outcomes and functional recovery is not well defined. The aim of this review was to assess the impact of postoperative aerobic exercise training, either alone or in conjunction with another exercise modality, on patients who have had surgery for intra-abdominal cancer.

**Methods:**

A literature search was performed of electronic journal databases. Eligible papers needed to report an outcome of aerobic capacity in patients older than 18 years of age, who underwent cancer surgery with curative intent and participated in an exercise programme (not solely ERAS) that included an aerobic exercise component starting at any point in the postoperative pathway up to 12 weeks.

**Results:**

Eleven studies were deemed eligible for inclusion consisting of two inpatient, one mixed inpatient/outpatient and eight outpatient studies. Meta-analysis of four outpatient studies, each reporting change in 6-min walk test (6MWT), showed a significant improvement in 6MWT with exercise (MD 74.92 m, 95% CI 48.52–101.31 m). The impact on health-related quality of life was variable across studies.

**Conclusion:**

Postoperative exercise confers benefits in improving aerobic function post surgery and can be safely delivered in various formats (home-based or group/supervised).

**Supplementary Information:**

The online version contains supplementary material available at 10.1007/s10151-023-02844-9.

## Introduction

Nearly half of all adults in the UK will develop cancer at some point during their lives [1]. Surgery remains the gold standard for achieving a curative outcome in many of these cases, especially for intra-abdominal cancers. Various prediction tools and preoperative assessment models such as the ColoRectal Physiological and Operative Severity Score for the enumeration of Mortality and morbidity (CR-POSSUM) score are used to try and appropriately triage patients who may need more intensive perioperative support, based on an established evidence base showing that physical fitness at the time of operation is strongly associated with improved postsurgical outcomes [2, 3]. In recent years, prehabilitation for cancer surgery has received increasing attention in both research and clinical spheres [4]. Designed to improve the functional status of patients prior to surgery (even within the time-sensitive period between cancer diagnosis) in order to improve postoperative outcomes, the supportive evidence for prehabilitation in patients with cancer is most commonly based around exercise training, although often with adjuvant multidisciplinary elements such as nutritional advice and/or psychological support [5, 6]. However, to date, there is little focus for clinicians on amalgamated evidence and therefore advisory body guidance about exercise rehabilitation for this particular cohort of patients. This is despite evidence that rehabilitation in other surgical cohorts significantly improves functional outcomes for patients [7, 8].

It is well known that the presence of cancer has a catabolic effect, with many patients presenting with systemic symptoms including skeletal muscle loss, weight loss, fatigue, and difficulty performing activities of daily living [9]. In those who are eligible for surgical resection with curative potential, reduced physical activity levels, often attributed to fatigue and weakness, can impact their ability to withstand the physical demands of this treatment [10]. In addition, when considering cancer as a disease of ageing (e.g. despite the increase in diagnoses in younger adults, the incidence of colorectal cancer rises sharply after the age of 50 years [11]), other age-associated conditions such as sarcopenia may also negatively impact physiological resilience for surgery [12].

Recognising the importance of optimal surgical recovery, not only for the patient but also for healthcare systems in terms of length of stay and associated costs, has led to the design and implementation of enhanced recovery after surgery (ERAS) programmes [13]. Providing targets for both patients and healthcare professionals, the primary aim of these programmes is to reduce the length of postoperative stay and complication rate [14]. A meta-analysis of randomised controlled trials (RCTs) assessing the effect of ERAS programmes on morbidity, complications and length of stay showed that they did shorten length of hospital stay without increasing rates of readmissions, although there was no difference in surgical complication rate [15].

Similar to prehabilitation regimes which cease at the point of surgery, ERAS programmes often stop at the point of hospital discharge. With little in the way of clear guidelines for what patients can aim to achieve after surgery, especially in patients with cancer, they are commonly provided with little clear instruction on what they should aim to do when at home until their follow-up appointment, which can often be many weeks later. UK government guidelines state that all healthy adults should aim to do either 75 min of vigorous exercise or 150 min of moderate exercise per week, with at least two resistance exercise sessions per week to promote whole-body health [16]. In patients with active cancer, aerobic exercise training, even at a vigorous intensity, has been shown to be both safe and effective for improving health-related outcomes (i.e. cardiorespiratory fitness, fatigue, patient-perceived fitness, and sleep) [17]. In addition, when combined with appropriate dietary intake (i.e. adequate protein), resistance exercise training has also been shown to improve muscle mass and function in various populations of patients with cancer [18–20]. However, bespoke guidelines for patients after cancer surgery are not available. As both cardiorespiratory and muscle function are each associated with favourable health outcomes, especially in older adults [20–22], the physiological benefits of exercise for this patient cohort are clear. In addition, the psychological benefits of exercise are also well established, an aspect of heightened importance for patients dealing with a cancer diagnosis and the impacts of treatment [23, 24].

Given the well-established benefits of perioperative exercise for patients with cancer, including a growing body of evidence for exercise-based prehabilitation yet a lack of tailored exercise advice for patients with intra-abdominal cancer postoperatively, the aim of this work was to review the current literature to determine if aerobic exercise training as rehabilitation, either alone or in conjunction with another exercise modality, (i) is feasible in the postoperative setting; (ii) confers any physiological benefits in terms of aerobic capacity; and (iii) has any significant effect on patients’ psychological well-being or health-related quality of life (HRQoL).

## Methods

### Study design

The review was registered on PROSPERO prior to literature searches (registration number CRD42021175427). Cohort studies, RCTs, and non-RCTs were included, with abstracts and case reports excluded. The Preferred Reporting Items for Systematic Reviews and Meta-Analyses (PRISMA) flow chart was used to assess papers for inclusion in the final review [25].

### Inclusion and exclusion criteria

Only studies of adult patients (aged 18 and over) diagnosed with an abdominal malignancy and who had undergone resectional surgery with curative intent were included. Full details on the inclusion and exclusion criteria are detailed in Table 1. All intra-abdominal cancers were included as the method of entry to the abdomen is similar and the focus of this review is the impact of rehabilitation on postsurgical recovery.

**Table 1:** Inclusion and exclusion criteria for article selection

Inclusion criteria
Adult patients over the age of 18 years with an abdominal malignancy
Patients undergoing any mode (i.e. open, laparoscopic, robotic, etc.) of resectional surgery with curative intent
Postoperative exercise programme (inpatient, outpatient or mixed) with an aerobic exercise training component
A reported outcome of cardiorespiratory fitness
Studies that compare either pre- and postoperative measures, or compare an exercise group to control
Exclusion criteria
Patients who have not undergone intra-abdominal surgery with curative intent
Palliative patients or those undergoing surgical resection for benign disease
Preoperative exercise only or studies that only compare prehabilitation to rehabilitation, with no reference to baseline changes within the two groups
Exercise programmes that start more than 12 weeks postoperatively
Qualitative only studies
Studies that assess the impact of an enhanced recovery after surgery (ERAS) protocol

### Search strategy and article selection

**Fig. 1:**
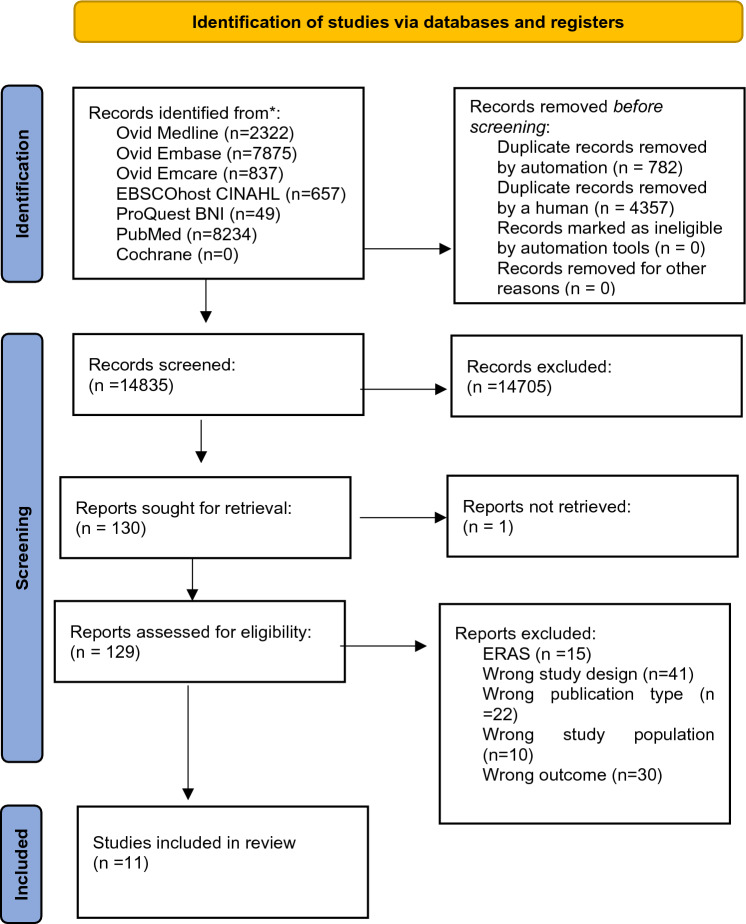
Preferred Reporting Items for Systematic Reviews and Meta-Analyses (PRISMA) flow chart showing the process of article identification and inclusion

A clinical librarian (ST) conducted searches of OVID Medline, OVID Embase, OVID Emcare, EBSCOhost CINAHL, ProQuest BNI, PubMed, and Cochrane databases (see Search Strategy in Appendix 1 of the supplementary material). All searches were run on 13th March 2023. Articles searched for were in any language and with no date restriction. Abstracts from the initial search results were filtered using Rayyan systematic review software [26] to exclude duplicates and identify papers to be further screened for inclusion. The process of article identification and exclusion is shown in Fig. 1.

### Outcomes

The primary outcome was a measure representing aerobic capacity, to determine if exercise rehabilitation elicited any physiological benefit. Other clinical outcomes included length of hospital stay, rates of postoperative complications, and postoperative morbidity and mortality. Patient-centred outcomes included BMI, HRQoL (via questionnaire) and markers of physical function such as 1-repetition maximum (1-RM) and 30-s chair stand. Outcomes related to feasibility included adherence and compliance of the exercise regimes.

### Quality assessment

Study quality in randomised trials was assessed using the Cochrane Collaboration’s tool for assessing risk of bias (RoB2) [27]. For non-randomised studies, the Risk Of Bias In Non-randomized Studies—of Interventions (ROBINS-I) tool was used [28].

### Data extraction and statistical analysis

Abstract screening was performed by one individual (MP) and rescreened in a blinded manner (TS), with differences resolved by consensus agreement.

Effect estimates are reported as mean differences (MD) with 95% confidence intervals (CI). As a result of inconsistent reporting of mean changes and change standard deviations (SD), these were calculated using formulae from the Cochrane Handbook [29]. A correlation coefficient of 0.7 was assumed between baseline and final values based on previous similar data [30]. Means were estimated from medians, and SD from range [31]. For outcomes with sufficient data, meta-analysis using a restricted maximum likelihood random-effects model was performed [32]. Statistical heterogeneity was assessed using the* I*^2^ statistic. GRADE was used to assess the certainty of evidence for the 6MWT [32] and all analyses were conducted using Stata Version 16 (StataCorp, College station, TX, USA).

## Results

### Included studies

Eleven studies were included: 6 RCTs, 1 pilot study, 1 retrospective cohort study and 3 feasibility trials [33–43]. Studies were conducted between 2014 and 2022, and all were published in the English language. The total number of patients across all studies was 734, with colorectal cancer the most prominent cancer type studied. Other cancer types included gastric, oesophageal and urological. Details of the included studies can be seen in Table 2.

**Table 2:** Included studies [33–37, 39–42, 62]

Study first author & year	Country	Study design	ERAS–type protocol as SOP?	Surgical approach *n* (%)	Total no. of participants	Intervention group no	Control group no.	Cancer type	Inpatient, outpatient or mixed	Primary outcome	Type of exercise	Length of exercise (weeks)	Location of exercise	Aerobic capacity outcome	QoL assessment tool
de Almeida (2017)	Brazil	Single blind RCT	Yes	Laparoscopic 24 (22%) Open 84 (78%)	108	54	54	Mixed abdominal	Inpatient	Inability to walk without human assistance at POD5 or hospital discharge	Aerobic, core, gait, isometric and isotonic	Until discharge	Ward-based	6MWT	EQ-5D-5L
Do (2022)	South Korea	Retrospective cohort	NES	Robotic 38 (64%) Open 21 (36%)	59	29	30	Oesophageal	Inpatient	Not specified	Aerobic, pulmonary rehab and resistance	Until discharge	Ward-based	6MWT	EORTC QLQ-C30
Cho (2018)	South Korea	Single arm interventional feasibility study	Yes	Laparoscopic 8 (40%) Robotic 12 (60%)	20	20	n/a	Gastric	Mixed	Feasibility	Aerobic and resistance	10	Mixed inpatient, outpatient supervised, and home-based	VO_2_ peak	EORTC QLQ-C30, EORTC QLQ-STO22
Simonsen (2020)	Denmark	Non-randomised controlled feasibility study	NES	Robotic assisted 2 (4%) Hybrid 7 (14%) Open 33 (67%)*	49	20	29	GOJ	Outpatient	Feasibility	Aerobic and resistance	12	Hospital-based supervised	Peak power output	FACT-E
Chang (2019)	Taiwan	RCT	NES	All open	88	44	44	Oesophageal	Outpatient	Quality of life	Aerobic	12	Home-based	6MWT, mean VO2 max	EORTC QLQ C30, EORTC QLQ-OES18
Gillis (2014)	Canada	Single blind RCT	Yes	Laparoscopic 72 (94) Open 5 (6%)	77	39	38	Colorectal	Outpatient	Functional exercise capacity (6MWT)	aerobic and resistance	8	Home-based	6MWT	SF-36, HADS
Porserud (2014)	Sweden	Single blind RCT	Yes	Open 18 (100%)	18	9	9	Urological (cystectomy)	Outpatient	Not specified	Aerobic mobility, strength and stretching	12	Group hospital-based	6MWT	SF-36
Mascherini (2020)	Spain	RCT	NES	Laparosopic 6 (100%)	6	3	3	Colorectal	Outpatient	Not specified	Aerobic and resistance	26	Mixed supervised and home-based	6MWT	n/a
Frawley (2020)	Australia	Non-randomised controlled	NES	Method of access NES	188	84	104	Mixed abdominopelvic	Outpatient	Feasibility	Aerobic and resistance	8	Supervised group at rehabilitation site	6MWT	ICIQ, IPAQ-SF, HADS, EORTC QLQ C-30
Carli (2020)	Canada	Single blind RCT	Yes	Open 23 (21%) MIO 87 (79%)	110	55	55	Colorectal	Outpatient	30-day complications	Aerobic and resistance	4	Hospital and home-based	6MWT	HADS, CHAMPS, SF-36
Nusca (2021)	Italy	Pilot	NES	Laparoscopic 11 (100%)	11	6	5	Colorectal	Outpatient	EORTC QLQ-C30 for QoL improvement	Aerobic and muscle strengthening	8	Hospital based supervised	6MWT	EORTC QLQ-C30, HADS

*RCT* randomised controlled trial, *SOP* standard operating procedure, *NES* not explicitly stated, *MIO* minimally invasive operation, *POD* postoperative day, *6MWT* 6-min walk test, *EQ-5D-5L* EuroQol- 5 Dimension, *VO2 peak* peak volume of oxygen consumed (during exercise), *EORTC QLQ-C30* European Organisation for Research and Treatment of Cancer Core Quality of Life questionnaire,* EORTC QLQ-STO22* European Organisation for Research and Treatment of Cancer Quality of Life questionnaire–Gastric Cancer Module,* GOJ* gastro-oesophageal junction,* SF-36* 36-Item Short Form Survey, *HADS* Hospital Anxiety and Depression Scale, *ICIQ* International Consultation on Incontinence Modular Questionnaires, *IPAQ-SF* International Physical Activity Questionnaire-Short Form, *CHAMPS* Community Healthy Activities Model Program for Seniors questionnaire, *QoL* quality of life, *ERAS *Enhanced recovery after surgery

*7 patients excluded as no surgery performed or tumour not resected

### Bias assessment

**Fig. 2:**
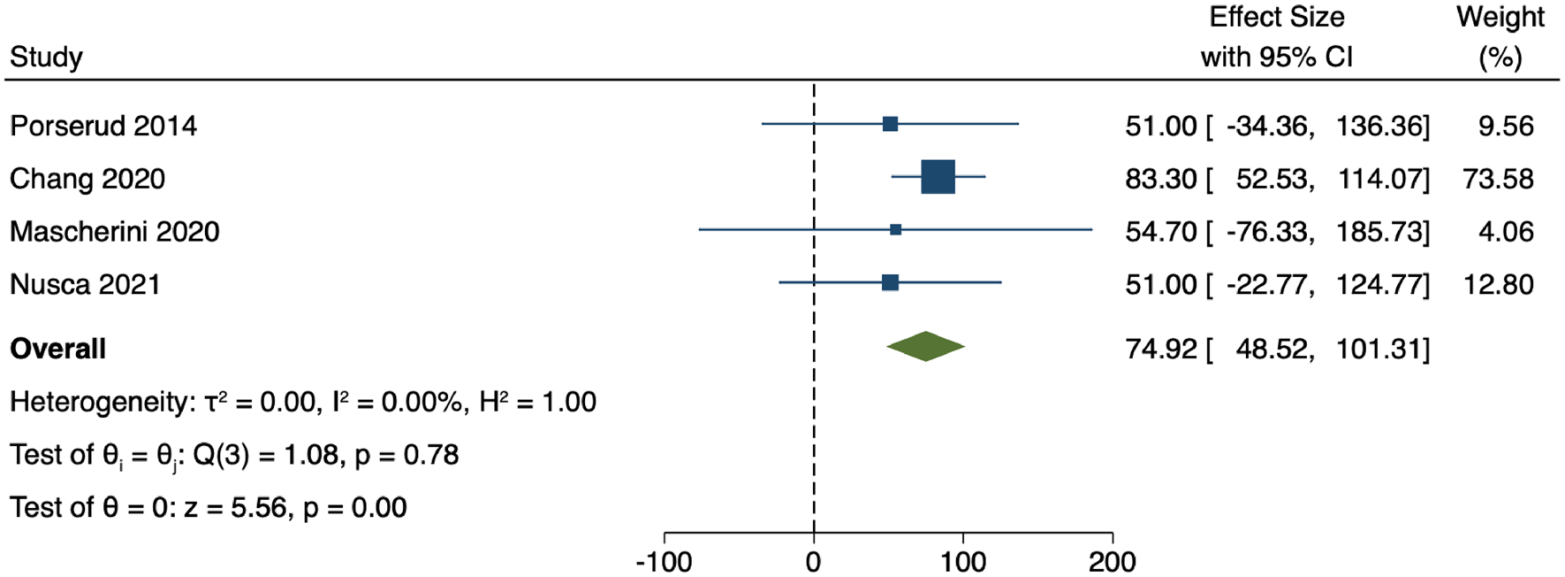
Forest plot showing the difference in 6-min walk test (6MWT) distance between exercise and control groups from 4 studies that employed 6MWT as an outpatient exercise outcome measure

Across all the studies eligible for inclusion in this review, risk of bias was elevated in non-controlled compared to controlled trials. The full results of this assessment are seen in Fig. 2. The overall GRADE certainty of evidence for the studies included in the meta-analysis of 6MWT is low. This is mainly due to the overall risk of bias, as one study was not a randomised controlled trial.

### Inpatient-based studies

Two studies had an aerobic outcome in patients undergoing a dedicated postoperative exercise programme prior to discharge [33, 43], with the majority of screened inpatient studies focussed on ERAS regimens to reduce hospital length of stay (LoS) without an outcome related to aerobic capacity. de Almeida et al*. *randomized 108 patients who had undergone major abdominal oncological surgery into an early mobilization (exercise) group (EX, *n* = 54) or standard postoperative care (CON, *n* = 54). The exercise protocol involved core, gait, isometric, isotonic and aerobic training. Patients underwent a baseline preoperative assessment, measuring thigh circumference and performing a 6-min walk test (6MWT), with 6MWT and HRQoL also assessed at postoperative day (POD) 5. The primary outcome for this study was ability to cross a room without human assistance postoperatively; 16.7% of patients were unable to cross the room unassisted in the EX group compared to 38.9% in CON (*p* = 0.010; relative risk (RR) 0.42, 95% CI 0.22–0.85). Although the EX group performed significantly better in the 6MWT compared to CON [212 m (56–299) vs. 66 m (0–228), *p* = 0.004], there was no significant different in LoS (EX 8 days (6–13) vs. CON 8 days (7–13), *p* = 0.25). Despite a lack of difference in LoS, the EX group did have better HRQoL scores (via the EQ-5D-5L index, which reports on mobility, self-care, usual activities, pain/discomfort and anxiety and depression) at POD5 compared to CON (0.71 (0.48–0.88) vs. 0.34 (90.19–0.73), *p* < 0.001). However, this benefit appeared to be short lived as there was no significant difference between the groups at POD30.

Do et al. introduced a new multimodal rehabilitation programme to replace an existing pulmonary rehabilitation regimen for a cohort of patients who underwent surgery for oesophageal cancer [43]. They compared QoL outcomes, 6MWT and other markers of physical function including 30-s chair stand test and grip strength, between the two groups. There were no significant differences between the two groups at baseline, including for surgery type and disease staging. They found significant within-group differences between pre and post surgery in left handgrip strength, 30-s chair stand and 6MWT (mean difference between pre- and postoperative 6MWT distance: multimodal rehabilitation versus pulmonary rehabilitation 73.1 ± 52.6 vs. 28.4 ± 14.3, *p* < 0.001, *d* = 1.15). The authors posited that a potential cause for the differences seen was the introduction of aerobic and resistance training to attenuate the effects of reduced physical function and to improve cardiorespiratory function, especially given the surgical approach often employed (through the chest wall).

### Mixed studies (inpatient and outpatient)

Only one study had a programme that started during inpatient stay and continued post discharge [38]. Most screened mixed studies were excluded as a result of no aerobic capacity outcome assessment. The majority of outcomes were related LoS, readmissions and/or complication rates. Cho et al. developed and piloted a postoperative exercise recovery programme for patients who had undergone either laparoscopic or robotic gastrectomy for gastric cancer, called PREP-GC. Twenty patients completed the programme following surgery, which started during their postoperative inpatient admission. The inpatient exercise component consisted of isokinetic exercises, stretches and walking, which continued for a week post discharge at home. For the subsequent 8 weeks, patients underwent a supervised aerobic and resistance exercise programme consisting of aerobic and stretch-based warm-up and cool-down movements and a variety of resistance exercises. The primary outcome for this study was incidence of adverse events during the exercise programme with feasibility also assessed by rates of adherence and compliance. All patients completed the exercise programme with no adverse events. The adherence and compliance rates were 95.2% and 80%, respectively. Eleven patients required minor modifications to the outpatient exercise programme, totalling 17 (0.6%) of the 2908 individual exercise components performed.

In terms of aerobic capacity, absolute VO_2_ peak increased (*p* < 0.001) after the exercise programme, returning, from an initial decrease postoperatively (*p* < 0.05), to levels numerically similar to preoperative levels (preoperative, 2.27 ± 6.18 L/min; postoperative, 1.80 ± 4.38 L/min; post PREP-GC, 2.16 ± 5.05 L/min). Other measures of physical function including 30-s chair stand and half-squat test also improved following the exercise programme compared to preoperative assessment.

As expected, HRQoL scores using the European Organization for Research and Treatment of Cancer Quality of Life Questionnaire-Stomach Cancer-Specific Module (EORTC QLQC30 and EORTC QLQ-STO22) were reduced in the period after surgery, but improved significantly following the PREP-GC exercise programme (*p* < 0.05), including in symptom-related domains such as fatigue, nausea and pain. Using the EORTC QLQ-C30, physical, social, cognitive and role functioning parameters were shown to decrease immediately after surgery before increasing during the postoperative period. Conversely, a sustained improvement in emotional functioning was shown, even during the immediate postoperative period. This improvement was perhaps attributable to the exercise programme given that this is at odds with what has been shown in previous studies that reported a sustained reduction in emotional functioning during the short-term (within 1 month) postoperative period [44–46].

### Outpatient-based studies

Eight studies had exercise programmes which started after hospital discharge to outpatient status [34–37, 40–42]. These interventions started between 0 and 11 weeks postoperatively and were between 4 and 12 weeks in duration.

#### Adherence and compliance

Six of the eight outpatient studies reported on adherence [29–31, 34, 35] and/or compliance [28, 36]. Of the six studies that did report compliance, four [35–37, 39] reported the attrition rate after the exercise programme had started (23%, range 7–45%), with attrition between randomisation and study completion slightly lower (21%, range 0–50%) on the basis of all six outpatient studies. Further details on compliance can be seen in Table 3.

**Table 3:** Exercise completion rate from included studies that employed outpatient exercise interventions [35–37, 39–41]

Study first author & year	Location of exercise	Exercise completion rate
Simonsen (2020)	Hospital-based	19 randomised to exercise group, 16 started programme, 13 finished 90.4% completion rate of aerobic exercise 75.5% completion rate of resistance exercise
Gillis (2014)	Home-based	44 randomised to exercise group, 42 started programme, 39 finished Postoperative compliance rates; mean % (SD): 0–4 weeks: prehab group 53% (30%), rehab group 31% (26%) 4–8 weeks: prehab group 53% (33%), rehab group 40% (31%)
Porserud (2014)	Group session; hospital-based	9 randomised to exercise group, 5 started programme, 4 finished 76% (67–95) attendance rate at group exercise training sessions
Frawley (2020)	Group sessions; rehabilitation site	84 randomised to exercise group, 75 finished 81% attended 85–100% of 16 scheduled training sessions 56% received scheduled telephone coaching sessions
Carli (2020)	Hospital and home-based	60 randomised to rehab exercise group, 55 included in intention-to-treat analysis, 30 finished
Nusca (2021)	Hospital-based	6 randomised to exercise group, 6 finished 100% exercise adherence rate (note enrolment rate of 29% for all eligible patients)

Chang et al. [34] did not document compliance rates

#### Aerobic outcomes

Of the eight studies included in the results, seven reported the 6MWT as one of their outcomes related to aerobic capacity [34, 36, 37, 40–42]. 6MWT has been shown to correlate with both aerobic capacity and functional performance [47, 48]. Studies by Carli et al. and Gillis et al. were excluded from this analysis as they were directly comparing groups having undergone prehabilitation versus rehabilitation with no control group [39, 40]. Frawley et al.’s study was excluded as there was no data available for the control group [37].

**Fig. 3:**
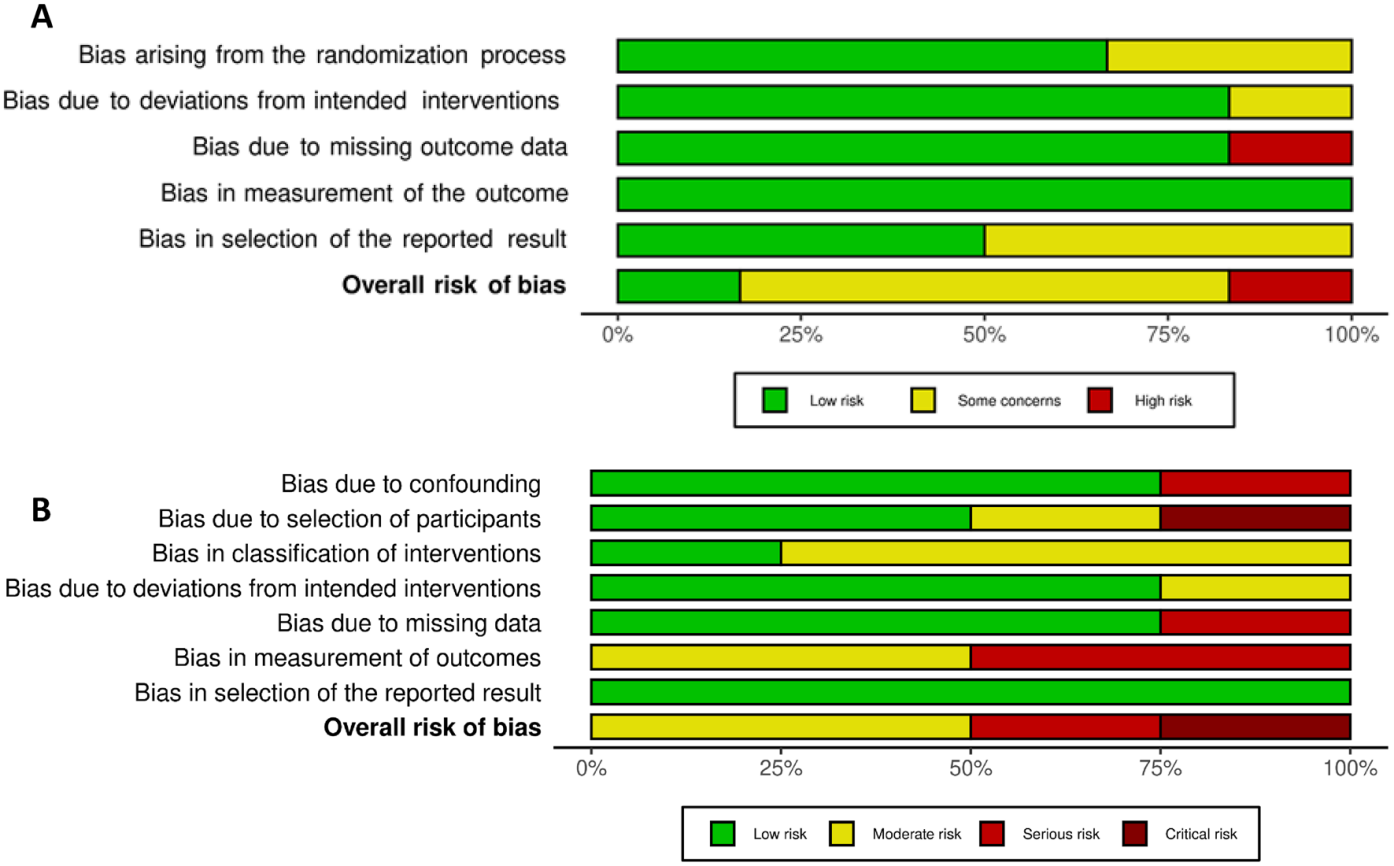
Result of bias assessment of the randomised controlled studies using ROB2 tool (**a**) and non-randomised studies using ROBINS-I tool (**b**)

Meta-analysis of the remaining four studies showed a significant increase in 6MWT distance in the intervention groups compared to the control groups (MD 74.92, 95% CI 48.52–101.31; *p* < 0.01) [34, 36, 41, 42] as seen in Fig. 3. There was no statistical heterogeneity between these studies (*I*^2^ = 0%).

Simonsen et al. used either a stationary bicycle or a treadmill to measure peak power output as their primary aerobic capacity outcome. As expected, there was a reduction in mean peak power output in the exercise group in the immediate postoperative period, but this returned to or improved from baseline by the end of exercise training in the intervention group. The control group did not undergo aerobic testing, limiting the inference of the impact of the exercise intervention on recovery.

#### Health-related quality of life

To assess changes in HRQoL a range of different validated questionnaires were used. All included studies assessed HRQoL except for Mascherini et al. The most commonly used questionnaire was the SF-36 followed by the EORTC-QLQ C30. Other questionnaires used included EORTC cancer-specific subsets, and the Hospital Anxiety and Depression Scale (HADS) questionnaire. A summary of HRQoL findings is presented in Table 4.

**Table 4:** Summary of HRQoL outcomes in the outpatient studies [34–36, 39–41]

Study first author & year	Questionnaire used	Outcome	
Simonsen (2020)	FACT-E	Total score at 7–14 months—no between group differences	
		Exercise group: significant improvement in total score at 7–14-month follow-up (within group) (i.e. post exercise programme); control: no significant change in total score	
Porserud (2014)	SF-36	Exercise group: Role physical—significant improvement in score at T2 (post exercise) assessment compared to pre-exercise (*P* = 0.031). No other significant difference seen at any other time point measured in either group	
Nusca (2021)	EORTC QLQ C30	Exercise group: significant difference seen in the following domains: physical functioning (PF2), cognitive functioning (CF) and fatigue (FA)	
	Domain:	End of exercise (2 months postoperatively):	4 months postoperatively:
	PF2	0.03	0.018
	CF	0.018	N/A
	FA	0.017	0.045
	HADS	No significant difference between groups at any time points in any domain	
Gillis (2014)	SF-36	No significant difference between groups at any time points in any domain; no within-group differences reported	
	HADS	No significant difference between groups at any time points in any domain; no within-group differences reported	
Carli (2020)	SF-36	No significant difference between groups at 4 weeks post surgery	
	HADS	No significant difference between groups at 4 weeks post surgery	
Chang (2020)	EORTC-QLQ-C30	Exercise group: significantly lower scores (less severe symptoms) for insomnia than controls at 3 months (*β* = − 12.81, 95% CI − 2.74, − 0.89, *p* < 0.05, respectively). Scores for nausea and vomiting were also significantly lower for the intervention than control groups at 3 and 6 months (*β* = − 12.62, 95% CI − 20.48, − 4.79, *p* < 0.01; and *β* = − 11.67, 95% CI − 20.77, − 2.57, *p* < 0.05, respectively)	
	EORTC-QLQ-OES18	At 3 months the intervention group had significantly lower scores for dysphagia than controls (*β* = − 12.56, 95% CI − 21.34, − 3.76, *p* < 0.01). Loss of taste was also significantly lower at 6 months (*β* = − 13.66, 95% CI − 2240, − 4.93, *p* < 0.01 respectively)	

*FACT-E* Functional Assessment of Cancer Therapy-oesophageal cancer QOL specific items, *EORTC QLQ-C30* European Organisation for Research and Treatment of Cancer Core Quality of Life Questionnaire, *EORTC QLQ-OES18* European Organisation for Research and Treatment of Cancer Quality of Life questionnaire-Oesophageal Cancer Module, *SF-36* 36-Item Short Form Survey,* HADS* Hospital Anxiety and Depression Scale

## Discussion

Given the known multiple benefits of exercise training for healthy adults [49, 50] and numerous different clinical cohorts [51, 52], it may seem obvious that exercise after surgery would confer both physical and psychological benefits to patients, as shown in this review. However, the magnitude of benefit is highly variable even across a relatively small number of studies and is likely multifactorial, involving factors such as format and length of exercise programme and method of delivery. Despite an evidence-based supposition [53, 54] and emerging direct evidence [55] for the benefits of exercise training in the postoperative period, there is still very little in the way of established guidance for patients or healthcare professionals pertaining to exercise in this phase of the journey of a patient with cancer. This may be due to the postoperative rehabilitation period falling between the purview of different healthcare professionals, i.e. physiotherapists rather than the surgical team. In addition to providing advice for those who are not educated in exercise prescription, such guidelines may also help with complex patient perceptions. Although some patients with cancer and associated healthcare practitioners do view exercise as a tool to help with both emotional and physical well-being, others may believe it to do “more harm than good”; however, this is most commonly not the case [56]. As can be seen from the studies included in this review, adverse event rates were very low in those completing postoperative exercise training.

Another consideration for exercising patients with cancer is the logistical burden of their diagnosis and treatment plan. Patients will likely already be faced with multiple cancer-related commitments (i.e. clinic visits) and as such exercise delivery method will likely contribute to patient adherence. For example, multiple trips to an external centre/hospital may reduce the rate of enrolment and/or compliance. For example, Frawley et al. used patients who were unwilling or unable to complete the exercise programme as their control group. Only 24% of patients approached consented to enrol on their exercise programme, with those in the control group living significantly further away from the rehabilitation site than the exercise group. Conversely, Gillis et al. delivered a home-based rehabilitation programme, in which 89% of eligible patients agreed to randomisation and only 3 out of 42 patients were lost to follow-up after the start of the programme. Although these findings suggest that home-based exercise may be favourable as a result of the logistical burden of ‘on-site’ exercise training, the impact of supervision must also be considered. If a home-based exercise programme is used, remote supervision using telehealth tools may be invaluable to help maintain compliance, such as in Chang et al., where a two-way informatics system encouraged communication between the healthcare team and patients [34].

In relation to optimal timing of intervention delivery, two studies included in this review compared prehabilitation to rehabilitation and showed inconsistent results. Carli et al. showed that there was no difference in recovery of walking capacity between the two groups at 4 weeks postoperatively, whereas Gillis et al. showed more favourable results from the prehabilitation group at 2 months post surgery (mean difference 45.4 m, 95% CI 13.9–77.0). There were, however, differences between these studies. Carli et al. had an older patient population (median age of rehab group 82, IQR 75–84) than Gillis et al. (mean age 66, SD 9.1) and there were also differences in the length of the training. The programme delivered by Carli et al. was 4 weeks, whereas Gillis et al. employed an 8-week programme. This suggests that a longer exercise programme may lead to a larger improvement; however, despite a relative wealth of recent data showing the positive impact that exercise prehabilitation can have on physical [57, 58], clinical [2] and psychological [24] outcomes for surgical patients with cancer, the mandated limited time frame (of less than 31 days) between decision to treat and operation for patients with cancer undergoing surgery with curative intent can limit the degree of possible improvement [59]. For example, 6 weeks high-intensity interval training (an exercise modality commonly employed in prehabilitation) has been shown to be needed to improve peak oxygen uptake in individuals age-matched to those most commonly presenting for colorectal cancer resection [60]. In addition, with its origin in anaesthetics, prehabilitation efforts also tend to have a focus on improving short-term clinical outcomes after surgery such as LoS, complication rate and 30/90-day mortality, rather than focusing on return to baseline QoL and/or activities of daily living. Conversely, postoperative rehabilitation exercise programmes can be delivered over a longer period of time and can also be adapted and/or extended until the patient reaches specific goals. This goal-setting approach may help to improve patient adherence and compliance, especially if the targets are developed in concordance with the patient [61]. Considering the benefits of both pre- and rehabilitation, one proposition is that for those patients who are both willing and able, both these intervention strategies could be used in tandem to prime patients to be resilient to the physiological insult of surgery* and* to help them return to their pre-illness activities and quality of life as quickly as possible.

This review does have limitations which need to be acknowledged. Firstly, studies which delivered exercise only as part of an ERAS programme were excluded as such programmes tend to be multi-faceted (i.e. including intraoperative targets) and often start preoperatively, and so may not give an accurate account of the value of exercise alone. This has likely impacted the number of studies eligible for inclusion in this review. Secondly, although all the scores used to determine QoL were obtained via well-validated questionnaires, that different questionnaires were used across studies prohibited meta-analysis. A consensus on the use of or development of one comprehensive questionnaire that can be used to assess QoL at various time points in the clinical pathway of a patient with cancer regardless of cancer type would be beneficial for future research. Thirdly, some of the studies had small sample sizes, including those in the meta-analysis of 6MWT and therefore this meta-analysis was heavily weighted. It should be noted that 6MWT was not the primary outcome for some of these studies, and as such they may not have been powered appropriately for this endpoint. There were also insufficient included studies to conduct assessment for publication bias or investigate heterogeneity.

## Conclusion

This review supports the development of formal exercise guidance for postoperative patients with cancer to aid their physical and psychological recovery, with questions around postoperative exercise being commonly asked by patients at surgical follow-up. This review suggests that exercise rehabilitation for these patients may be valuable not only in improving physiological parameters but also in improving psychosocial functioning. However, how this would be delivered in a pragmatic cost-effective way is yet not clear. Only once the evidence base in this field is established, e.g. via a multicentre, prospective RCT as an example of the high-quality research required in this space, can the true benefit of postoperative exercise be realised, allowing development and implementation of formalised guidelines in a multidisciplinary manner for patients with intra-abdominal cancer facing surgery.

### Supplementary Information

Below is the link to the electronic supplementary material.

Supplementary file1 (PDF 478 KB)

## Data Availability

All data supporting the findings of this study are available within the paper and its Supplementary Information, or via access to the papers quoted.
