# Supplementary material for: A systematic review of the impact of postoperative aerobic exercise training in patients undergoing surgery for intra-abdominal cancers
Source: Tech Coloproctol. 2023 Aug 7;27(12):1169–81. doi: 10.1007/s10151-023-02844-9 (PMC10638144; doi:10.1007/s10151-023-02844-9)
Supplement: Supplementary file 1 — Supplementary file1 (PDF 478 KB) [file 10151_2023_2844_MOESM1_ESM.pdf]

Appendix 1: Search Strategy

Search Strategy – Ovid Medline

**Search Terms**
("post op*" or "post-op*" or "postop*" or "post operative" or "post-operative" or "postoperative" or (after ADJ4 surg*) or (post ADJ4 surg*) or (follow* ADJ4 surg*) or inpatient* or "after care" or "after-care" or "aftercare" or "after discharge" or "post discharge" or outpatient*).mp. OR exp AFTERCARE/ OR exp INPATIENTS/ OR exp OUTPATIENTS/ OR exp "POSTOPERATIVE CARE"/ OR exp "POSTOPERATIVE PERIOD"/<br><br>**AND**<br><br>(aerobic OR (muscle ADJ4 train*) OR "enhanced recovery" OR exercise* OR "strength training" OR sport* OR weightlifting OR "weight* training" OR "weight* bearing strengthening" OR (resistance ADJ4 train*) OR (weight* ADJ4 lift)).mp OR exp EXERCISE/ OR exp "EXERCISE THERAPY"/ OR exp "WEIGHT LIFTING"/ OR exp "RESISTANCE TRAINING"/ OR exp RUNNING/ OR exp SWIMMING/ OR exp WALKING/ OR exp SPORTS/<br><br>**AND**<br>((abdo* or anal or bladder or bowel or cervi* or colon or colorectal or endometr* or gastr* or gynae* or intestin* or liver or ovar* or prostat* or rectal or stomach or urolog* or uter* or pancrea*) ADJ4 (neoplasm* or tumor* or tumour* or cancer*)).mp OR exp "ABDOMINAL NEOPLASMS"/ OR exp "COLONIC NEOPLASMS"/ OR exp "COLORECTAL NEOPLASMS"/ OR exp "ENDOMETRIAL NEOPLASMS"/ OR exp "GASTROINTESTINAL NEOPLASMS"/ OR "GASTROINTESTINAL STROMAL TUMORS"/ OR "LIVER NEOPLASMS" OR exp "INTESTINAL NEOPLASMS"/ OR exp NEOPLASMS/ OR exp "OVARIAN NEOPLASMS"/ OR exp "PERITONEAL NEOPLASMS"/ OR exp "PROSTATIC NEOPLASMS"/ OR exp "STOMACH NEOPLASMS"/ OR exp "URINARY BLADDER NEOPLASMS"/ OR exp

"UROLOGIC NEOPLASMS"/ OR exp "UTERINE NEOPLASMS"/ OR exp "UTERINE CERVICAL NEOPLASMS"/ OR exp "PANCREATIC NEOPLASMS"/

AND

(laparotom* OR laparoscop* OR keyhole OR "robotic surg*" OR tumour ADJ4 excis* OR tumor ADJ4 excis* OR surg*).mp OR exp SURGICAL PROCEDURES, OPERATIVE/ OR exp "HAND-ASSISTED LAPAROSCOPY"/ OR exp "LAPAROTOMY"/ OR exp "LAPAROSCOPY"/ OR exp "MINIMALLY INVASIVE SURGICAL PROCEDURES"/ OR exp "ROBOTIC SURGICAL PROCEDURES"/

Search Strategy – Ovid Embase

Search Terms

("post op*" or "post-op*" or "postop*" or "post operative" or "post-operative" or "postoperative" or (after adj2 surg*) or (post adj2 surg*) or (follow* adj2 surg*) or inpatient* or "after care" or "after-care" or "aftercare" or "after discharge" or "post discharge" or outpatient*).mp. OR exp AFTERCARE/ OR exp "HOSPITAL PATIENT"/ OR exp OUTPATIENT/ OR exp "OUTPATIENT CARE"/ OR exp "POSTOPERATIVE PERIOD"/

AND

(aerobic OR "muscle ADJ3 train*" OR "enhanced recovery" OR exercise* OR "strength training" OR sport* OR weightlifting OR "weight* training" OR "weight* bearing strengthening" OR (resistance adj4 train*) OR "weight* ADJ4 lift").mp OR exp "MUSCLE EXERCISE"/ OR exp "LOW INTENSITY EXERCISE"/ OR exp "MODERATE INTENSITY EXERCISE"/ OR exp "DYNAMIC EXERCISE"/ OR exp "AEROBIC EXERCISE"/ OR exp "ANAEROBIC EXERCISE"/ OR exp "ISOTONIC EXERCISE"/ OR exp "ISOMETRIC EXERCISE"/ OR exp "HIGH INTENSITY EXERCISE"/ OR exp "ISOKINETIC EXERCISE"/ OR exp "SQUATTING (EXERCISE)"/ OR exp EXERCISE/ OR exp "ARM EXERCISE"/ OR exp "AQUATIC EXERCISE"/ OR exp "STRETCHING EXERCISE"/ OR exp "TREADMILL

EXERCISE"/ OR exp "EXERCISE INTENSITY"/ OR exp "STATIC EXERCISE"/ OR exp "EXERCISE TOLERANCE"/ OR exp "LEG EXERCISE"/ OR exp KINESIOTHERAPY/ OR exp "WEIGHT LIFTING"/ OR exp "RESISTANCE TRAINING"/ OR exp RUNNING/ OR exp SWIMMING/ OR exp WALKING/ OR exp "NORDIC WALKING"/ OR exp "MUSCLE TRAINING"/ OR "LACROSSE (SPORT)"/ OR exp ENDURANCE SPORT/ OR exp "SQUASH (SPORT)"/ OR BALL SPORT/ OR exp SPORT/ OR exp "CRICKET (SPORT)"/ OR exp AERONAUTICAL SPORT/ OR exp "CROSS TRAINING (SPORT)"/ OR exp "SURFING (WATER SPORT)"/ OR "SPORTS AND SPORT RELATED PHENOMENA"/ OR exp DISABLED SPORT/ OR exp WATER SPORT/ OR exp COLLISION SPORT/ OR exp RACQUET SPORT/ OR exp TEAM SPORT/ OR exp "FENCING (SPORT)"/ OR exp WINTER SPORT/ OR exp NON CONTACT SPORT/ OR exp COMBAT SPORT/ OR exp "SAILING (WATER SPORT)"/ OR exp CONTACT SPORT/ OR exp WHEELCHAIR SPORT/

AND

((abdo* or anal or bladder or bowel or cervi* or colon or colorectal or endometr* or gastr* or gynae* or intestin* or liver or ovar* or prostat* or rectal or stomach or urolog* or uter* or pancrea*) adj3 (neoplasm* or tumor* or tumour* or cancer*)).mp OR exp "ABDOMINAL CANCER"/ OR exp "ABDOMEN METASTASIS CELL LINE"/ OR exp "ABDOMINAL TUMOR"/ OR exp "COLON CANCER"/ OR exp "COLON CARCINOMA"/ OR exp "COLON TUMOR"/ OR exp "COLORECTAL CANCER"/ OR exp "COLORECTAL TUMOR"/ OR exp "DIGESTIVE SYSTEM CANCER"/ OR exp "ENDOMETRIUM CANCER"/ OR exp "ENDOMETRIUM TUMOR"/ OR "GASTROINTESTINAL STROMAL TUMOR"/ OR exp "GASTROINTESTINAL TUMOR"/ OR exp "INTESTINE CANCER"/ OR exp "INTESTINE TUMOR"/ OR exp "LIVER CANCER"/ OR exp "LIVER TUMOR"/ OR "OVARY CANCER"/ OR "OVARY TUMOR"/ OR exp "PERITONEUM CANCER"/ OR exp "PERITONEUM TUMOR"/ OR exp "PROSTATE CANCER"/ OR exp "PROSTATE TUMOR"/ OR exp "STOMACH CANCER"/ OR exp "STOMACH TUMOR"/ OR "BLADDER CANCER"/ OR "BLADDER TUMOR"/ OR "URINARY TRACT CANCER"/ OR "URINARY TRACT TUMOR"/ OR "UTERUS CANCER"/ OR exp "UTERINE CERVIX CANCER"/ OR exp "UTERINE CERVIX TUMOR"/ OR exp "UTERUS

CARCINOMA"/ OR exp "RECTUM CANCER"/ OR exp "RECTUM TUMOR"/ OR exp "RECTUM CARCINOMA"/ OR exp COLON CANCER/ OR exp COLON CARCINOMA/ OR exp RECTUM CANCER/ OR exp RECTUM TUMOR/ OR exp COLON TUMOR/ OR exp RECTUM CARCINOMA/ OR exp PANCREAS CANCER/ OR exp PANCREAS TUMOR/

AND

((laparotom* OR laparoscop* OR keyhole OR "robotic surg*" OR (tumour adj3 excis*) OR (tumor adj3 excis*) OR surg*)).mp OR exp SURGERY/ OR exp "LAPAROSCOPY"/ OR exp "GASLESS LAPAROSCOPY"/ OR exp "HAND-ASSISTED LAPAROSCOPY"/ OR exp SURGERY/ OR exp LAPAROSCOPIC SURGERY/ or exp LAPAROENDOSCOPIC SINGLE SITE SURGERY/ OR exp "LAPAROTOMY"/ OR exp "MINIMALLY INVASIVE SURGERY"/ OR exp "ROBOT ASSISTED SURGERY"/ OR exp STOMACH SURGERY/ OR exp BLADDER SURGERY/ OR exp PANCREAS SURGERY/ OR exp RECTUM SURGERY/ OR exp CANCER SURGERY/ OR exp UROLOGIC SURGERY/ OR exp ABDOMINAL SURGERY/ OR exp UTERINE TUBE SURGERY/ OR exp ANUS SURGERY/ OR exp PROSTATE SURGERY/ OR exp URINARY TRACT SURGERY/ OR exp GYNECOLOGIC SURGERY/ OR exp COLORECTAL SURGERY/ OR exp GENERAL SURGERY/ exp COLON SURGERY/ OR exp INTESTINE SURGERY/ OR exp UTERUS SURGERY/ OR exp GASTROINTESTINAL SURGERY/ OR exp LIVER SURGERY/ or exp URETHRA SURGERY/ or exp SPLEEN SURGERY/ OR exp BILIARY TRACT SURGERY/ or exp KIDNEY SURGERY/ OR exp TRANSANAL ENDOSCOPIC SURGERY/ or exp URETER SURGERY/

Search Strategy – Ovid Emcare

Search Terms
("post op*" or "post-op*" or "postop*" or "post operative" or "post-operative" or "postoperative" or (after adj4 surg*) or (post adj4 surg*) or (follow* adj4 surg*) or inpatient* or "after care" or "after-care" or "aftercare" or "after discharge" or "post

discharge" or outpatient*).mp. OR exp AFTERCARE/ OR exp "HOSPITAL PATIENT"/ OR exp OUTPATIENT/ OR exp "OUTPATIENT CARE"/ OR exp "POSTOPERATIVE PERIOD"/

AND

(aerobic OR "muscle adj4 train*" OR "enhanced recovery" OR exercise* OR "strength training" OR sport* OR weightlifting OR "weight* training" OR "weight* bearing strengthening" OR (resistance adj4 train*) OR "weight* adj4 lift").mp OR exp "MUSCLE EXERCISE"/ OR exp "LOW INTENSITY EXERCISE"/ OR exp "MODERATE INTENSITY EXERCISE"/ OR exp "DYNAMIC EXERCISE"/ OR exp "AEROBIC EXERCISE"/ OR exp "ISOTONIC EXERCISE"/ OR exp "ISOMETRIC EXERCISE"/ OR exp "HIGH INTENSITY EXERCISE"/ OR exp "ISOKINETIC EXERCISE"/ OR exp "SQUATTING (EXERCISE)"/ OR exp EXERCISE/ OR exp "ARM EXERCISE"/ OR exp "AQUATIC EXERCISE"/ OR exp "STRETCHING EXERCISE"/ OR exp "TREADMILL EXERCISE"/ OR exp "EXERCISE INTENSITY"/ OR exp "STATIC EXERCISE"/ OR exp "EXERCISE TOLERANCE"/ OR exp "LEG EXERCISE"/ OR exp KINESIOTHERAPY/ OR exp "WEIGHT LIFTING"/ OR exp "RESISTANCE TRAINING"/ OR exp RUNNING/ OR exp SWIMMING/ OR exp WALKING/ OR exp "NORDIC WALKING"/ OR exp "MUSCLE TRAINING"/

AND

((abdo* or anal or bladder or bowel or cervi* or colon or colorectal or endometr* or gastr* or gynae* or intestin* or liver or ovar* or prostat* or rectal or stomach or urolog* or uter* or pancrea*) adj4 (neoplasm* or tumor* or tumour* or cancer*)).mp OR exp "ABDOMINAL CANCER"/ OR exp "ABDOMEN METASTASIS CELL LINE"/ OR exp "ABDOMINAL TUMOR"/ OR exp "COLON CANCER"/ OR exp "COLON CARCINOMA"/ OR exp "COLON TUMOR"/ OR exp "COLORECTAL CANCER"/ OR exp "COLORECTAL TUMOR"/ OR exp "DIGESTIVE SYSTEM CANCER"/ OR exp "ENDOMETRIUM CANCER"/ OR exp "ENDOMETRIUM TUMOR"/ OR "GASTROINTESTINAL STROMAL TUMOR"/ OR exp "GASTROINTESTINAL TUMOR"/ OR exp "INTESTINE CANCER"/ OR exp "INTESTINE TUMOR"/ OR exp

"LIVER CANCER"/ OR exp "LIVER TUMOR"/ OR "OVARY CANCER"/ OR "OVARY TUMOR"/ OR exp "PERITONEUM CANCER"/ OR exp "PERITONEUM TUMOR"/ OR exp "PROSTATE CANCER"/ OR exp "PROSTATE TUMOR"/ OR exp "STOMACH CANCER"/ OR exp "STOMACH TUMOR"/ OR "BLADDER CANCER"/ OR "BLADDER TUMOR"/ OR "URINARY TRACT CANCER"/ OR "URINARY TRACT TUMOR"/ OR "UTERUS CANCER"/ OR exp "UTERINE CERVIX CANCER"/ OR exp "UTERINE CERVIX TUMOR"/ OR exp "UTERUS CARCINOMA"/ OR exp "RECTUM CANCER"/ OR exp "RECTUM TUMOR"/ OR exp "RECTUM CARCINOMA"/ OR exp "PANCREAS CANCER"/ OR exp "PANCREAS TUMOR"/

AND

((laparotom* OR laparoscop* OR keyhole OR "robotic surg*" OR (tumour adj4 excis*) OR (tumor adj4 excis*) OR surg*)).mp OR exp SURGERY/ OR exp "LAPAROSCOPY"/ OR exp "GASLESS LAPAROSCOPY"/ OR exp "HAND-ASSISTED LAPAROSCOPY"/ OR exp SURGERY/ OR exp LAPAROSCOPIC SURGERY/ or exp LAPAROENDOSCOPIC SINGLE SITE SURGERY/ OR exp "LAPAROTOMY"/ OR exp "MINIMALLY INVASIVE SURGERY"/ OR exp "ROBOT ASSISTED SURGERY"/ OR exp STOMACH SURGERY/ OR exp BLADDER SURGERY/ OR exp PANCREAS SURGERY/ OR exp RECTUM SURGERY/ OR exp CANCER SURGERY/ OR exp UROLOGIC SURGERY/ OR exp ABDOMINAL SURGERY/ OR exp UTERINE TUBE SURGERY/ OR exp ANUS SURGERY/ OR exp PROSTATE SURGERY/ OR exp URINARY TRACT SURGERY/ OR exp GYNECOLOGIC SURGERY/ OR exp COLORECTAL SURGERY/ OR exp GENERAL SURGERY/ exp COLON SURGERY/ OR exp INTESTINE SURGERY/ OR exp UTERUS SURGERY/ OR exp GASTROINTESTINAL SURGERY/ OR exp LIVER SURGERY/ or exp URETHRA SURGERY/ or exp SPLEEN SURGERY/ OR exp BILIARY TRACT SURGERY/ or exp KIDNEY SURGERY/ OR exp TRANSANAL ENDOSCOPIC SURGERY/ or exp URETER SURGERY/

Search Strategy – EBSCOhost CINAHL

Search Terms
("post op*" or "post-op*" or "postop*" or "post operative" or "post-operative" or "postoperative" or (after N4 surg*) or (post N4 surg*) or (follow* N4 surg*) or inpatient* or "after care" or "after-care" or "aftercare" or "after discharge" or "post discharge" or outpatient*) OR (MH "After Care") OR (MH "Inpatients") OR (MH "Outpatients") OR (MH "Postoperative Care+") OR (MH "Postoperative Period")<br><br>**AND**<br><br>(aerobic OR "muscle N4 train*" OR "enhanced recovery" OR exercise* OR "strength training" OR sport* OR weightlifting OR "weight* training" OR "weight* bearing strengthening" OR (resistance N4 train*) OR "weight* N4 lift") OR (MH "Exercise+") OR (MH "Resistance Training") OR (MH "Abdominal Exercises") OR (MH "Therapeutic Exercise") OR (MH "Exercise Intensity") OR (MH "Group Exercise") OR (MH "Extreme Sports") OR (MH "Sport Specific Training") OR (MH "Aquatic Sports+") OR (MH "Aeronautical Sports") OR (MH "Winter Sports+") OR (MH "Wheelchair Sports") OR (MH "Sports+") OR (MH "Athletic Training+") OR (MH "Body Building") OR (MH "Bowling") OR (MH "Contact Sports+") OR (MH "Cycling") OR (MH "Endurance Sports") OR (MH "Fencing") OR (MH "Golf") OR (MH "Gymnastics") OR (MH "Handball") OR (MH "Martial Arts") OR (MH "Mountaineering") OR (MH "Racquet Sports+") OR (MH "Rock Climbing") OR (MH "Running+") OR (MH "Skating+") OR (MH "Skiing+") OR (MH "Sports, Disabled+") OR (MH "Sports Participation") OR (MH "Target Sports+") OR (MH "Team Sports+") OR (MH "Weight Lifting") OR (MH "Archery") OR (MH "Snow Skiing+") OR (MH "Cross Country Skiing") OR (MH "Water Skiing") OR (MH "Ice Skating") OR (MH "Skateboarding") OR (MH "Jogging") OR (MH "Running, Distance") OR (MH "Sprinting") OR (MH "Tennis") OR (MH "Boxing") OR (MH "Football") OR (MH "Rugby") OR (MH "Wrestling") OR (MH "Diving") OR (MH "Scuba Diving") OR (MH "Snorkeling") OR (MH "Rowing") OR (MH "Swimming") OR (MH "Walking+") OR (MH "Nordic Walking") OR (MH "Dancing+") OR (MH "Exercise+") OR (MH "Abdominal Exercises") OR (MH "Aerobic Exercises+") OR (MH "Aerobic Dancing") OR (MH "Aquatic Exercises") OR (MH "Jumping") OR (MH "Lower Extremity Exercises") OR (MH "High-Intensity Interval Training") OR (MH "Group Exercise")

OR (MH "Endurance Training") OR (MH "Anaerobic Exercises") OR (MH "Muscle Strengthening+") OR (MH "Isokinetic Exercises") OR (MH "Isometric Exercises") OR (MH "Isotonic Exercises") OR (MH "Resistance Training") OR (MH "Pilates") OR (MH "Upper Extremity Exercises+") OR (MH "Arm Exercises") OR (MH "Horseback Riding")

AND

((abdo* or anal or bladder or bowel or cervi* or colon or colorectal or endometr* or gastr* or gynae* or intestin* or liver or ovar* or prostat* or rectal or stomach or urolog* or uter* or pancrea*) N4 (neoplasm* or tumor* or tumour* or cancer*)) OR exp "ABDOMINAL NEOPLASMS"/ OR exp "COLONIC NEOPLASMS"/ OR exp "COLORECTAL NEOPLASMS"/ OR "ENDOMETRIAL NEOPLASMS"/ OR exp "GASTROINTESTINAL NEOPLASMS"/ "/ OR exp "LIVER NEOPLASMS" OR exp "INTESTINAL NEOPLASMS"/ OR exp "OVARIAN NEOPLASMS"/ OR exp "PERITONEAL NEOPLASMS"/ OR exp "PROSTATIC NEOPLASMS"/ OR "STOMACH NEOPLASMS"/ OR "BLADDER NEOPLASMS"/ OR exp "UROLOGIC NEOPLASMS"/ OR exp "UTERINE NEOPLASMS"/ OR exp "CERVIX NEOPLASMS"/ OR exp "PANCREATIC NEOPLASMS"/

AND

((laparotom* OR laparoscop* OR keyhole OR "robotic surg*" OR (tumour N4 excis*) OR (tumor N4 excis*) OR surg*)) OR (MH "Surgery, Operative+") OR "LAPAROTOMY"/ OR "LAPAROSCOPY"/ OR exp "MINIMALLY INVASIVE PROCEDURES"/ OR "ROBOTIC SURGICAL PROCEDURES"/ OR "SURGERY, LAPAROSCOPIC"/

Search Strategy – ProQuest BNI

**Search Terms**

((NOFT("post op*" or "post-op*" or "postop*" or "post operative" or "post-operative" or "postoperative" or (after NEAR/4 surg*) or (post NEAR/4 surg*) or (follow* NEAR/4 surg*) or inpatient* or "after care" or "after-care" or "aftercare" or "after discharge" or "post discharge" or outpatient*) OR MAINSUBJECT.EXACT("Inpatient care") OR MAINSUBJECT.EXACT("Patients") OR MAINSUBJECT.EXACT("Postoperative period"))

AND

(NOFT(aerobic OR "muscle NEAR/4 train*" OR "enhanced recovery" OR exercise* OR "strength training" OR sport* OR weightlifting OR "weight* training" OR "weight* bearing strengthening" OR (resistance NEAR/4 train*) OR "weight* NEAR/4 lift") OR MAINSUBJECT.EXACT("Exercise") OR MAINSUBJECT.EXACT("Isometric exercise") OR MAINSUBJECT.EXACT("Strength training") OR MAINSUBJECT.EXACT("Sports") OR MAINSUBJECT.EXACT("Running") OR MAINSUBJECT.EXACT("Swimming") OR MAINSUBJECT.EXACT("Walking") OR MAINSUBJECT.EXACT("Weightlifting"))

AND

(NOFT((abdo* or anal or bladder or bowel or cervi* or colon or colorectal or endometr* or gastr* or gynae* or intestin* or liver or ovar* or prostat* or rectal or stomach or urolog* or uter* or pancrea*) NEAR/4 (neoplasm* or tumor* or tumour* or cancer*)) OR MAINSUBJECT.EXACT("Colorectal cancer") OR MAINSUBJECT.EXACT("Endometrial cancer") OR MAINSUBJECT.EXACT("Liver cancer") OR MAINSUBJECT.EXACT("Ovarian cancer") OR MAINSUBJECT.EXACT("Prostate cancer") OR MAINSUBJECT.EXACT("Stomach cancer") OR MAINSUBJECT.EXACT("Bladder cancer") OR MAINSUBJECT.EXACT("Uterine cancer") OR MAINSUBJECT.EXACT("Cervical cancer") OR MAINSUBJECT.EXACT("Pancreatic cancer"))

AND

(NOFT (laparotom* OR laparoscop* OR keyhole OR "robotic surg*" OR tumour NEAR/4 excis* OR tumor NEAR/4 excis* OR surg*) OR MAINSUBJECT.EXACT("Abdominal surgery") OR MAINSUBJECT.EXACT("Cancer surgery") OR MAINSUBJECT.EXACT("Gastrointestinal surgery") OR MAINSUBJECT.EXACT("Gynaecological surgery") OR MAINSUBJECT.EXACT("Laparoscopic surgery") OR MAINSUBJECT.EXACT("Surgery") OR MAINSUBJECT.EXACT("Laparotomy")))

Search Strategy – PubMed

Search Terms

("post op*" or "post-op*" or "postop*" or "post operative" or "post-operative" or "postoperative" or (after surg*) or (post surg*) or (follow* surg*) or inpatient* or "after care" or "after-care" or "aftercare" or "after discharge" or "post discharge" or outpatient*) [All Fields] OR AFTERCARE OR INPATIENTS OR OUTPATIENTS OR exp "POSTOPERATIVE CARE" OR "POSTOPERATIVE PERIOD" [MeSH Terms]

AND

(aerobic OR "muscle train*" OR "enhanced recovery" OR exercise* OR "strength training" OR sport* OR weightlifting OR "weight training" OR "weight bearing strengthening" OR (resistance train*) OR "weight lift") [All Fields] OR EXERCISE OR "EXERCISE THERAPY" OR exp "WEIGHT LIFTING" OR "RESISTANCE TRAINING" OR RUNNING OR SWIMMING OR WALKING [MeSH Terms]

AND

((abdo* or anal or bladder or bowel or cervi* or colon or colorectal or endometr* or gastr* or gynae* or intestin* or liver or ovar* or prostat* or rectal or stomach or urolog* or uter* or pancrea*) and (neoplasm* or tumor* or tumour* or cancer*))[All Fields] OR "ABDOMINAL NEOPLASMS" OR "COLONIC NEOPLASMS" OR exp

"COLORECTAL NEOPLASMS" OR "ENDOMETRIAL NEOPLASMS" OR "GASTROINTESTINAL NEOPLASMS" OR "GASTROINTESTINAL STROMAL TUMORS" OR "LIVER NEOPLASMS" OR "INTESTINAL NEOPLASMS" OR NEOPLASMS OR "OVARIAN NEOPLASMS" OR "PERITONEAL NEOPLASMS" OR "PROSTATIC NEOPLASMS" OR "STOMACH NEOPLASMS" OR "URINARY BLADDER NEOPLASMS" OR "UROLOGIC NEOPLASMS" OR "UTERINE NEOPLASMS" OR "UTERINE CERVICAL NEOPLASMS" OR "PANCREATIC NEOPLASMS" [MeSH Terms]

AND

(laparotom* OR laparoscop* OR keyhole OR "robotic surg*" OR tumour excis* OR tumor excis* OR surg*) [All Fields] OR "HAND-ASSISTED LAPAROSCOPY" OR "LAPAROTOMY" OR "LAPAROSCOPY" OR "MINIMALLY INVASIVE SURGICAL PROCEDURES" OR "ROBOTIC SURGICAL PROCEDURES" [MeSH Terms]

Auto-translated by PubMed into:

("post op*"[All Fields] OR "post op*"[All Fields] OR "postop*"[All Fields] OR "post-operative"[All Fields] OR "post-operative"[All Fields] OR "postoperative"[All Fields] OR ("after"[All Fields] AND "surg*"[All Fields]) OR ("post"[All Fields] AND "surg*"[All Fields]) OR ("follow*"[All Fields] AND "surg*"[All Fields]) OR "inpatient*"[All Fields] OR "after-care"[All Fields] OR "after-care"[All Fields] OR "aftercare"[All Fields] OR "after discharge"[All Fields] OR "post discharge"[All Fields] OR "outpatient*"[All Fields] OR ((("aftercare"[MeSH Terms] OR "aftercare"[All Fields] OR ("inpatient s"[All Fields] OR "inpatients"[MeSH Terms] OR "inpatients"[All Fields] OR "inpatient"[All Fields]) OR ("outpatient s"[All Fields] OR "outpatients"[MeSH Terms] OR "outpatients"[All Fields] OR "outpatient"[All Fields]) OR "exp"[All Fields]) AND "POSTOPERATIVE CARE"[All Fields]) OR "POSTOPERATIVE PERIOD"[All Fields])) AND ("aerobic"[All Fields] OR "aerobically"[All Fields] OR "bacteria, aerobic"[MeSH Terms] OR ("bacteria"[All Fields] AND "aerobic"[All Fields]) OR "aerobic bacteria"[All Fields] OR "aerobe"[All Fields] OR "aerobes"[All Fields] OR "exercise"[MeSH Terms] OR "exercise"[All Fields] OR "aerobics"[All Fields] OR "muscle train*"[All Fields] OR "enhanced recovery"[All Fields] OR "exercise*"[All Fields] OR "strength training"[All Fields] OR "sport*"[All Fields] OR ("weightlifter"[All Fields] OR "weightlifters"[All Fields] OR "weightlifting"[All Fields]) OR "weight training"[All Fields] OR "weight bearing strengthening"[All Fields] OR (("resist"[All Fields] OR "resistance"[All Fields] OR "resistances"[All Fields] OR "resistant"[All Fields] OR "resistants"[All Fields] OR "resisted"[All Fields] OR "resistence"[All Fields] OR "resistences"[All Fields] OR "resistent"[All Fields] OR

"resistibility"[All Fields] OR "resisting"[All Fields] OR "resistive"[All Fields] OR "resistively"[All Fields] OR "resistivities"[All Fields] OR "resistivity"[All Fields] OR "resists"[All Fields]) AND "train*"[All Fields]) OR "weight lift"[All Fields] OR ((("exercise"[MeSH Terms] OR "exercise"[All Fields] OR "exercises"[All Fields] OR "EXERCISE THERAPY"[MeSH Terms] OR ("exercise"[All Fields] AND "therapy"[All Fields]) OR "EXERCISE THERAPY"[All Fields] OR "exercise s"[All Fields] OR "exercised"[All Fields] OR "exerciser"[All Fields] OR "exercisers"[All Fields] OR "exercising"[All Fields] OR "EXERCISE THERAPY"[All Fields] OR "exp"[All Fields]) AND "WEIGHT LIFTING"[All Fields]) OR "RESISTANCE TRAINING"[All Fields] OR ("running"[MeSH Terms] OR "running"[All Fields] OR "runnings"[All Fields]) OR ("swimming"[MeSH Terms] OR "swimming"[All Fields] OR "swims"[All Fields]) OR ("walked"[All Fields] OR "walking"[MeSH Terms] OR "walking"[All Fields] OR "walks"[All Fields]))) AND ((("abdo*"[All Fields] OR "anal"[All Fields] OR ("bladder s"[All Fields] OR "urinary bladder"[MeSH Terms] OR ("urinary"[All Fields] AND "bladder"[All Fields]) OR "urinary bladder"[All Fields] OR "bladder"[All Fields] OR "bladders"[All Fields]) OR ("bowel s"[All Fields] OR "bowell"[All Fields] OR "intestines"[MeSH Terms] OR "intestines"[All Fields] OR "bowel"[All Fields] OR "bowels"[All Fields]) OR "cervi*"[All Fields] OR ("colon"[MeSH Terms] OR "colon"[All Fields] OR "colonic"[All Fields] OR "colons"[All Fields] OR "colon s"[All Fields] OR "colonal"[All Fields] OR "colonically"[All Fields] OR "colonitis"[All Fields]) OR "colorectal"[All Fields] OR "endometr*"[All Fields] OR "gastr*"[All Fields] OR "gynae*"[All Fields] OR "intestin*"[All Fields] OR ("liver"[MeSH Terms] OR "liver"[All Fields] OR "livers"[All Fields] OR "liver s"[All Fields]) OR "ovar*"[All Fields] OR "prostat*"[All Fields] OR ("administration, rectal"[MeSH Terms] OR ("administration"[All Fields] AND "rectal"[All Fields]) OR "rectal administration"[All Fields] OR "rectal"[All Fields]) OR ("stomach"[MeSH Terms] OR "stomach"[All Fields] OR "stomachs"[All Fields] OR "stomach s"[All Fields] OR "stomachal"[All Fields] OR "stomaches"[All Fields]) OR "urolog*"[All Fields] OR "uter*"[All Fields] OR "pancrea*"[All Fields]) AND ("neoplasm*"[All Fields] OR "tumor*"[All Fields] OR "tumour*"[All Fields] OR "cancer*"[All Fields])) OR ((("ABDOMINAL NEOPLASMS"[All Fields] OR "COLONIC NEOPLASMS"[All Fields] OR "exp"[All Fields]) AND "COLORECTAL NEOPLASMS"[All Fields]) OR "ENDOMETRIAL NEOPLASMS"[All Fields] OR "GASTROINTESTINAL NEOPLASMS"[All Fields] OR "GASTROINTESTINAL STROMAL TUMORS"[All Fields] OR "LIVER NEOPLASMS"[All Fields] OR "INTESTINAL NEOPLASMS"[All Fields] OR ("neoplasm s"[All Fields] OR "neoplasms"[MeSH Terms] OR "neoplasms"[All Fields] OR "neoplasm"[All Fields]) OR "OVARIAN NEOPLASMS"[All Fields] OR "PERITONEAL NEOPLASMS"[All Fields] OR "PROSTATIC NEOPLASMS"[All Fields] OR "STOMACH NEOPLASMS"[All Fields] OR "URINARY BLADDER NEOPLASMS"[All Fields] OR "UROLOGIC NEOPLASMS"[All Fields] OR "UTERINE NEOPLASMS"[All Fields] OR "UTERINE CERVICAL NEOPLASMS"[All Fields] OR "PANCREATIC NEOPLASMS"[All Fields])) AND ("laparotom*"[All Fields] OR "laparoscop*"[All Fields] OR ("keyhole"[All Fields] OR "keyholes"[All Fields]) OR "robotic surg*"[All Fields] OR (("cysts"[MeSH Terms] OR "cysts"[All Fields] OR "cyst"[All Fields] OR "neurofibroma"[MeSH Terms] OR "neurofibroma"[All Fields] OR

"neurofibromas"[All Fields] OR "tumor s"[All Fields] OR "tumoral"[All Fields] OR "tumorous"[All Fields] OR "tumour"[All Fields] OR "neoplasms"[MeSH Terms] OR "neoplasms"[All Fields] OR "tumor"[All Fields] OR "tumour s"[All Fields] OR "tumoural"[All Fields] OR "tumourous"[All Fields] OR "tumours"[All Fields] OR "tumors"[All Fields]) AND "excis*"[All Fields]) OR (("cysts"[MeSH Terms] OR "cysts"[All Fields] OR "cyst"[All Fields] OR "neurofibroma"[MeSH Terms] OR "neurofibroma"[All Fields] OR "neurofibromas"[All Fields] OR "tumor s"[All Fields] OR "tumoral"[All Fields] OR "tumorous"[All Fields] OR "tumour"[All Fields] OR "neoplasms"[MeSH Terms] OR "neoplasms"[All Fields] OR "tumor"[All Fields] OR "tumour s"[All Fields] OR "tumoural"[All Fields] OR "tumourous"[All Fields] OR "tumours"[All Fields] OR "tumors"[All Fields]) AND "excis*"[All Fields]) OR "surg*"[All Fields] OR ("HAND-ASSISTED LAPAROSCOPY"[All Fields] OR "LAPAROTOMY"[All Fields] OR "LAPAROSCOPY"[All Fields] OR "MINIMALLY INVASIVE SURGICAL PROCEDURES"[All Fields] OR "ROBOTIC SURGICAL PROCEDURES"[All Fields]))

Search Strategy – Cochrane

Search Terms
((("post op*" OR "post-op*" OR "postop*" OR "post operative" OR "post-operative" OR "postoperative" OR (after NEAR/4 surg*) OR (post NEAR/4 surg*) OR (follow* NEAR/4 surg*) OR inpatient* OR "after care" OR "after-care" OR "aftercare" OR "after discharge" OR "post discharge" OR outpatient*):ti,ab,kw OR [mh aftercare] OR [mh inpatients] OR [mh outpatients] OR [mh "postoperative care"] OR [mh "postoperative period"]) AND ((aerobic OR "muscle NEAR/4 train*" OR "enhanced recovery" OR exercise* OR "strength training" OR sport* OR weightlifting OR "weight* training" OR "weight* bearing strengthening" OR (resistance NEAR/4 train*) OR "weight* NEAR/4 lift"):ti,ab,kw OR [mh exercise] OR [mh "exercise therapy"] OR [mh "weight lifting"] OR [mh "resistance training"] OR [mh running] OR [mh swimming] OR [mh walking] OR [mh sports]) AND (((abdo* OR anal OR bladder OR bowel OR cervi* OR colon OR colorectal OR endometr* OR gastr* OR gynae* OR intestin* OR liver OR ovar* OR prostat* OR rectal OR stomach OR urolog* OR uter* OR pancrea*) NEAR/4 (neoplasm* OR tumor* OR tumour* OR cancer*)):ti,ab,kw OR [mh "abdominal neoplasms"] OR [mh "colonic neoplasms"] OR [mh "colorectal neoplasms"] OR [mh "endometrial neoplasms"] OR [mh "gastrointestinal neoplasms"] OR [mh "gastrointestinal stromal tumors"] OR [mh "liver neoplasms"] OR [mh "intestinal neoplasms"] OR [mh "ovarian neoplasms"] OR

[mh "peritoneal neoplasms"] OR [mh "prostatic neoplasms"] OR [mh "stomach neoplasms"] OR [mh "urinary bladder neoplasms"] OR [mh "urologic neoplasms"] OR [mh "uterine neoplasms"] OR [mh "uterine cervical neoplasms"] OR [mh "pancreatic neoplasms"]) AND ((laparotom* OR laparoscop* OR keyhole OR "robotic surg*" OR tumour NEAR/4 excis* OR tumor NEAR/4 excis* OR surg*):ti,ab,kw OR [mh surgical procedures, operative] OR [mh "hand-assisted laparoscopy"] OR [mh "laparotomy"] OR [mh "laparoscopy"] OR [mh "minimally invasive surgical procedures"] OR [mh "robotic surgical procedures"]))
